# Supplementary figures and images for: Expansion of a neural crest gene signature following ectopic MYCN expression in sympathoadrenal lineage cells in vivo
Source: PLoS One. 2024 Sep 18;19(9):e0310727. doi: 10.1371/journal.pone.0310727 (PMC11410271; doi:10.1371/journal.pone.0310727)

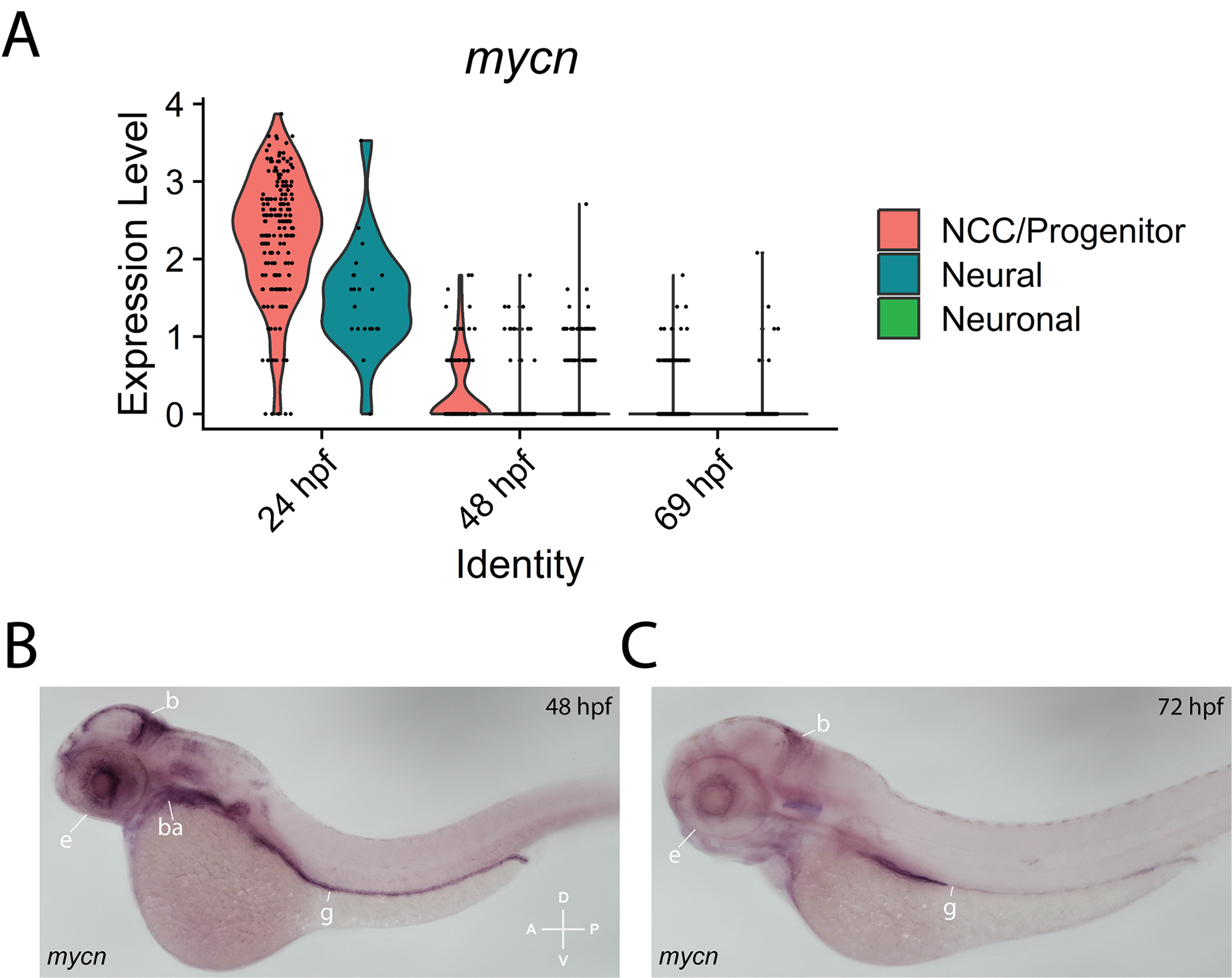

Supplement: S1 Fig — A) Violin plot showing expression of mycn is restricted to progenitor and NCC populations during early timepoints. B,C) representative images of whole mount in situ hybridization against mycn at 48 hpf (B) and 72 hpf (B) Anterior is shown to the left. A (anterior), P (posterior), D (dorsal), V (ventral) axes shown in lower right corner. b = developing brain, ba = developing branchial arches, g = developing gut, e = developing eye. (TIF) [file pone.0310727.s001.tif]

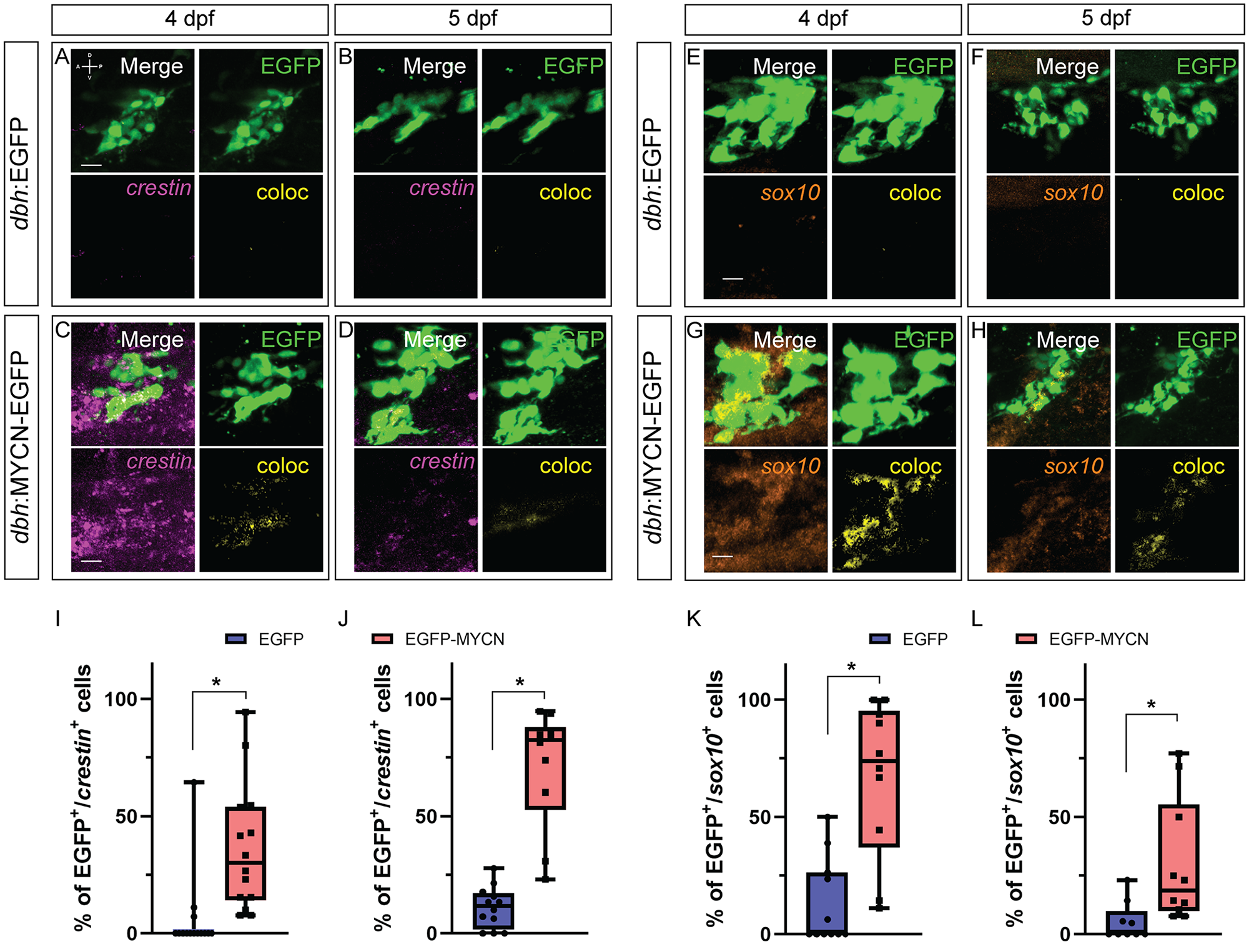

Supplement: S2 Fig — A,B,C,D) representative confocal images of SCG from dbh:EGFP (A,B) and dbh:EGFP-MYCN (C,D) larvae with WICHCR against crestin and EGFP. E,F,G,H) representative confocal images of SCG from dbh:EGFP (E,F) and dbh:EGFP-MYCN (G,H) larvae with WICHCR against sox10 and EGFP. Markers: EGFP (green), crestin (magenta), and coloc channel (yellow). A (anterior), P (posterior), D (dorsal), V (ventral) axes shown in upper left corner. Scale bars = 10 μm. I-L) Percentage of EGFP+ or EGFP-MYCN+ cells that also express crestin (I,J) or sox10 (K,L) at 4 dpf (I,K), and 5 dpf (J,L). For crestin 4 dpf EGFP n = 14, EGFP-MYCN n = 14; for crestin 5 dpf EGFP n = 12, EGFP-MYCN n = 10. For sox10 4 dpf EGFP n = 11, EGFP-MYCN n = 10; for sox10 5 dpf EGFP n = 9, EGFP-MYCN n = 10. For all graphs * denotes P<0.05. (TIF) [file pone.0310727.s002.tif]

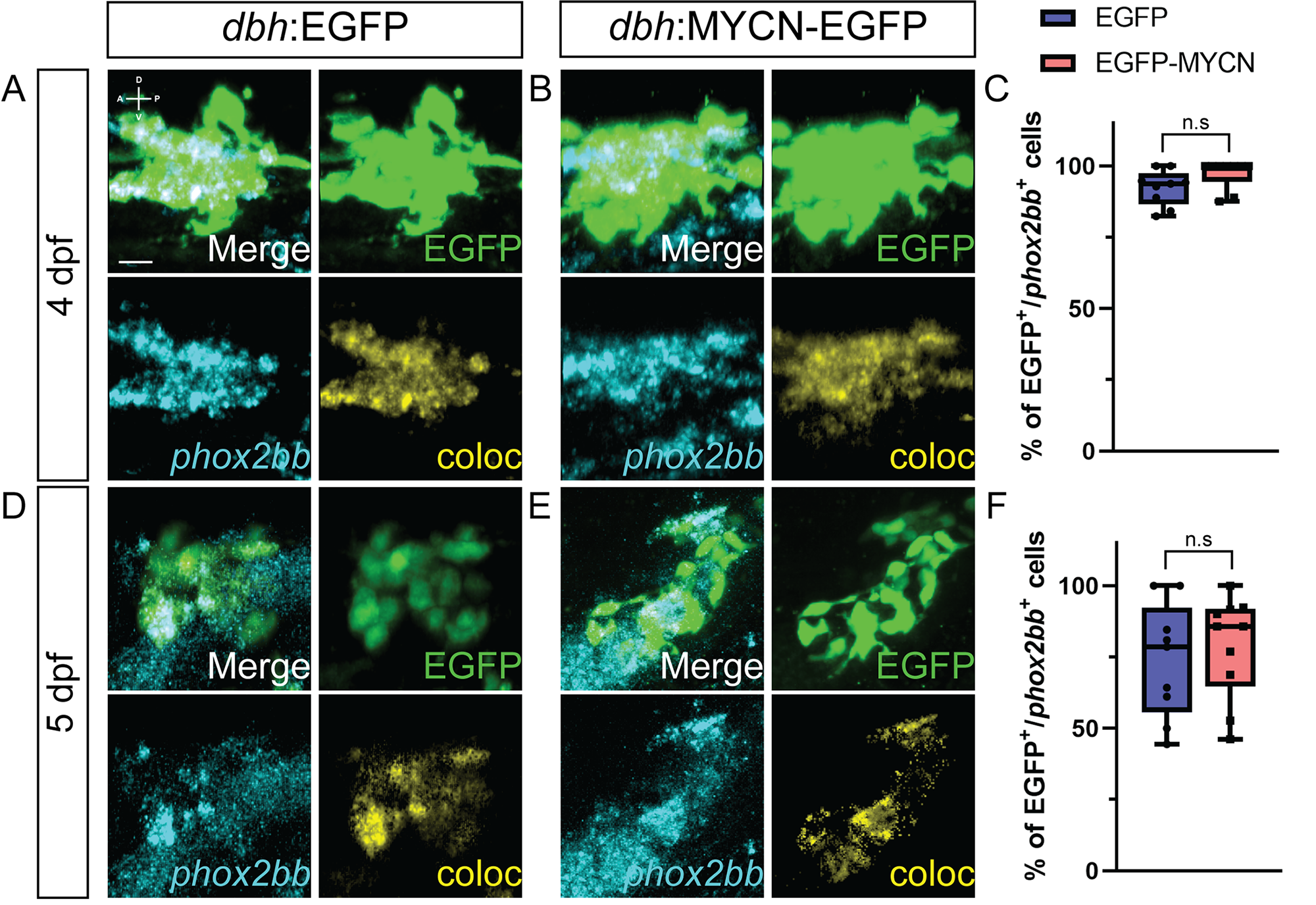

Supplement: S3 Fig — A,B,D,E) representative confocal images of SCG from dbh:EGFP (A,D) and dbh:EGFP-MYCN (B,E) larvae with WICHCR against phox2bb and EGFP. Markers: EGFP (green), phox2bb (cyan), and coloc channel (yellow). A (anterior), P (posterior), D (dorsal), V (ventral) axes shown in upper left corner. Scale bars = 10 μm. C,F) Percentage of EGFP+ or EGFP-MYCN+ cells that also express phox2bb at 4 dpf (C) and 5 dpf (F). For 4 dpf EGFP n = 9, EGFP-MYCN n = 9; for 5 dpf EGFP n = 9, EGFP-MYCN n = 10. For all graphs n.s. denotes non-significant (P>0.05). (TIF) [file pone.0310727.s003.tif]

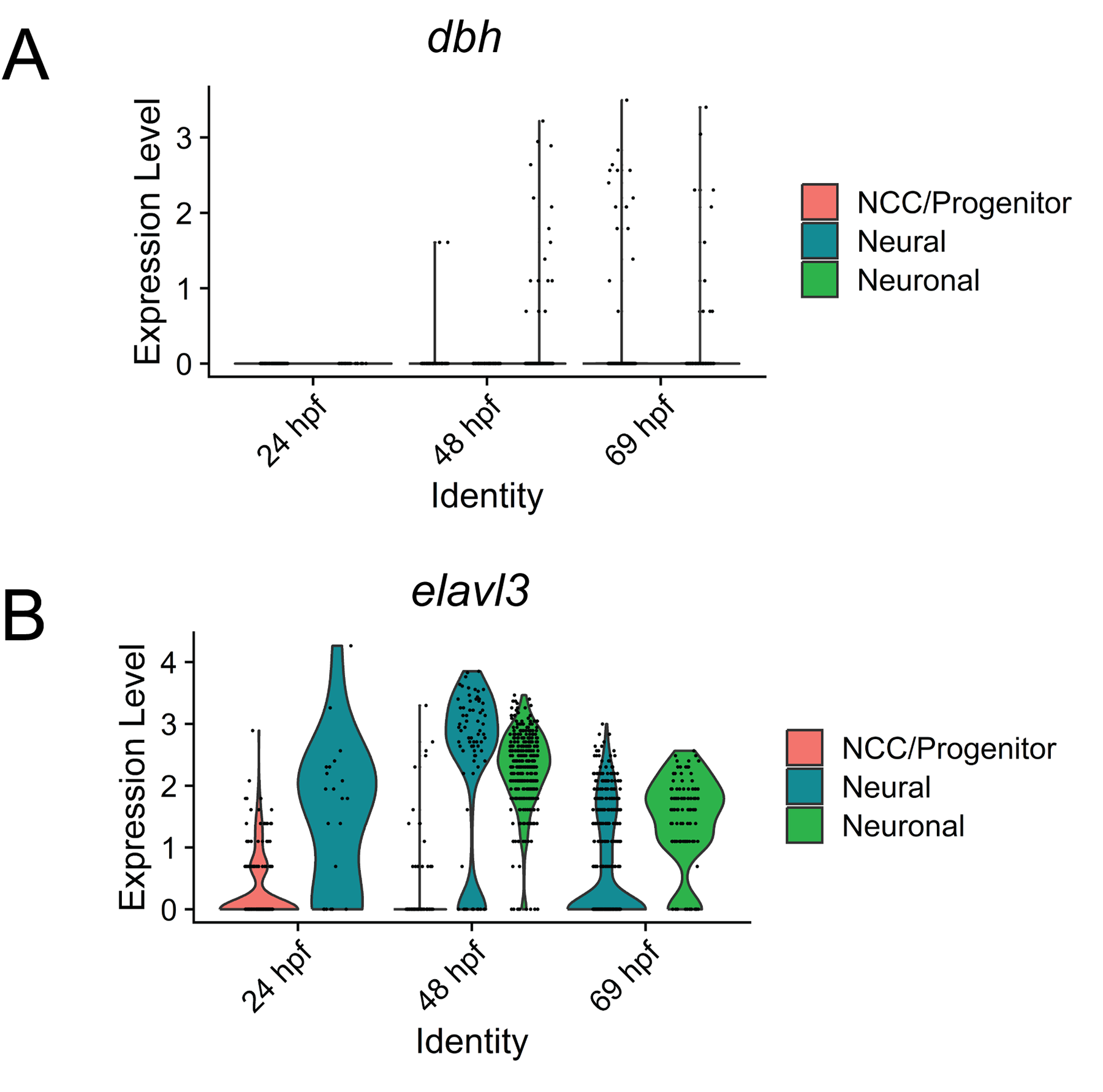

Supplement: S4 Fig — A,B) Violin plots depicting dbh (A) and elavl3 (B) expression from single cell datasets of sox10-derived cells at 24, 48, and 69 hpf, as described in methods. (TIF) [file pone.0310727.s004.tif]

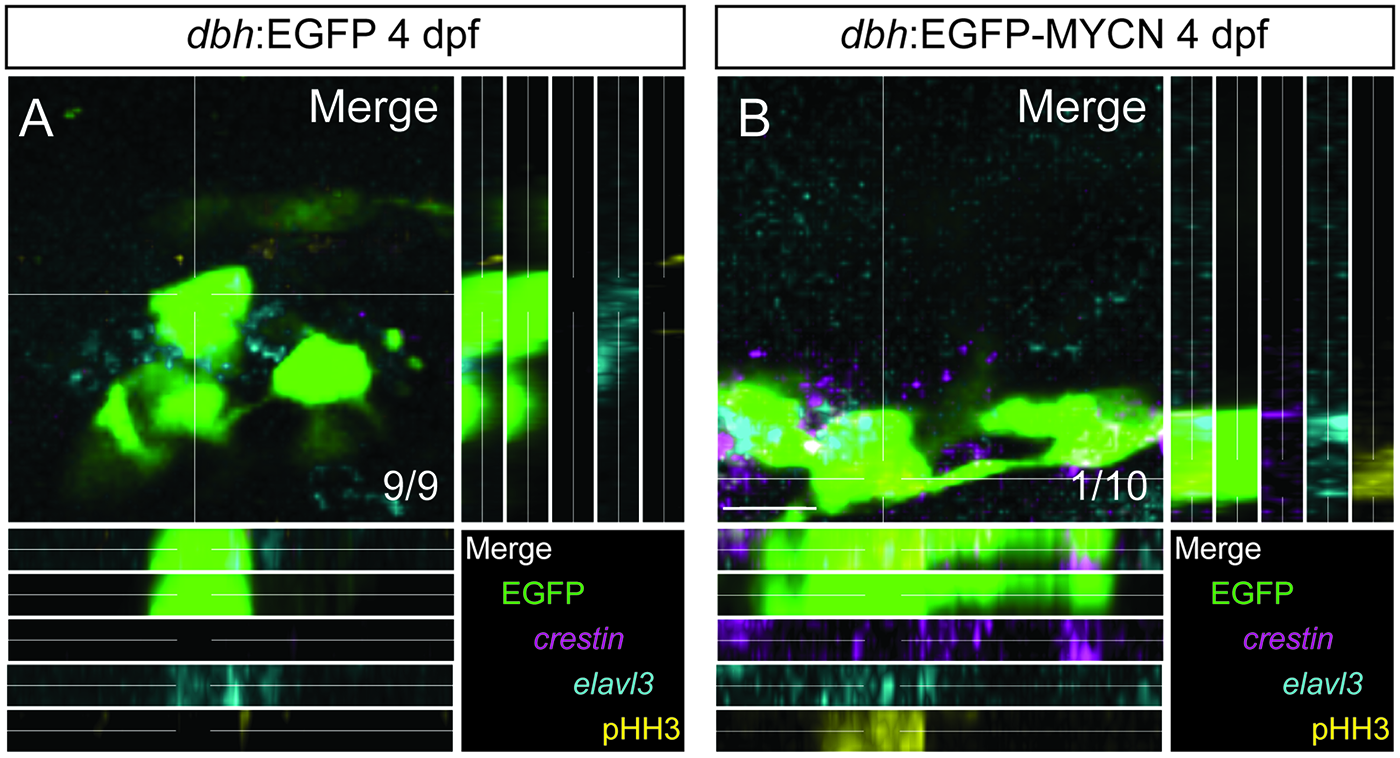

Supplement: S5 Fig — A,B) Images from sections through the developing SCG in dbh:EGFP (A) or dbh:EGFP-MYCN (B) larvae at 4 dpf. WICHCR performed using probes against crestin, elavl3, and with antibodies against EGFP and phosphorylated Histone H3 (pHH3). Proliferating SAP cells (pHH3+) were not detected in the SCG of either conditions. Only one MYCN-overexpressing larvae presented a proliferating (pHH3+/crestin+/elavl3+) cell. Markers: EGFP (green), crestin (magenta), elavl3 (cyan), and pHH3 (yellow). Scale bar = 10 μm. (TIF) [file pone.0310727.s005.tif]

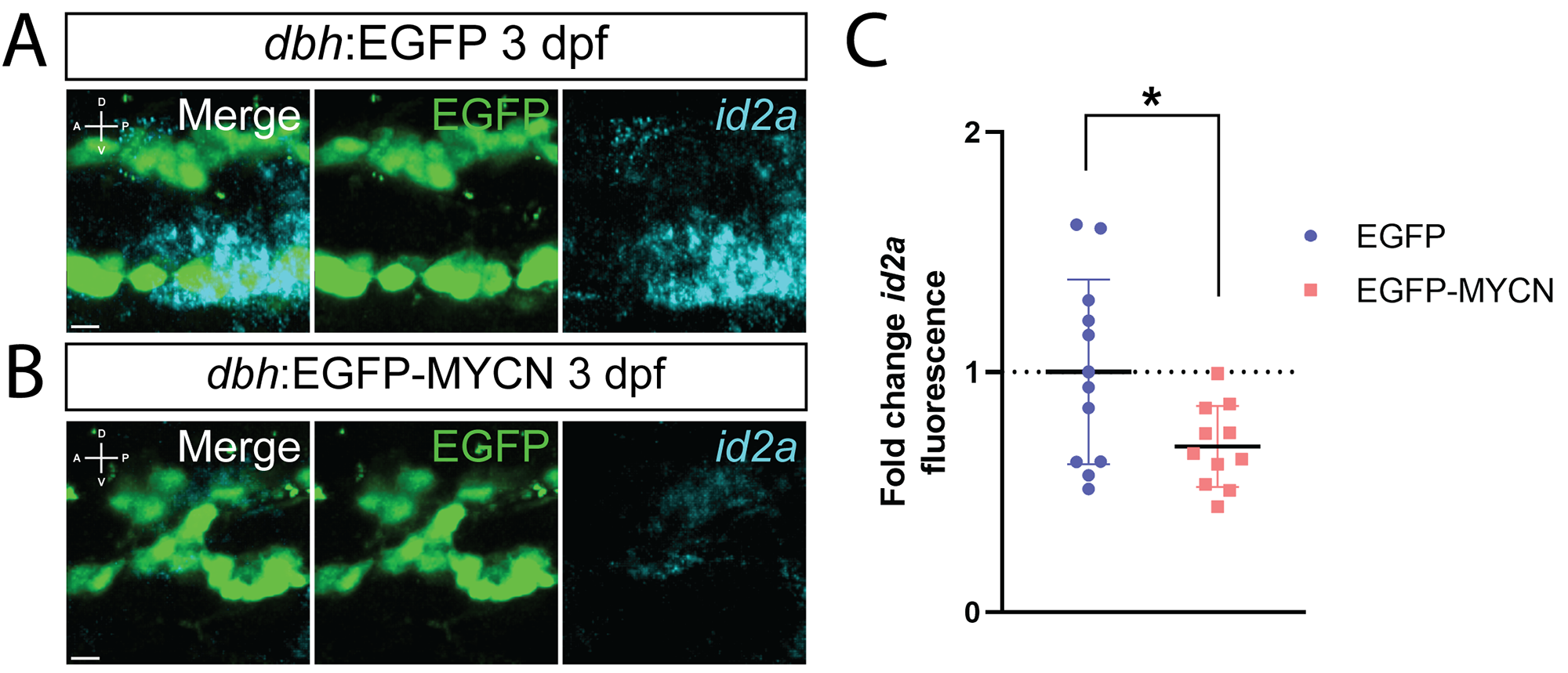

Supplement: S6 Fig — A,B) Confocal images of the developing SCG at 3 dpf in dbh:EGFP (A) or dbh:EGFP-MYCN (B) larvae. Markers: EGFP (green), id2a (cyan). A (anterior), P (posterior), D (dorsal), V (ventral) axes shown in upper left corner. Scale bars = 7 μm. C) Mean id2a fluorescence intensity quantified in the SCG and normalized to dbh:EGFP average intensity at 3 dpf. For EGFP n = 12, EGFP-MYCN n = 11. * P<0.05. (TIF) [file pone.0310727.s006.tif]

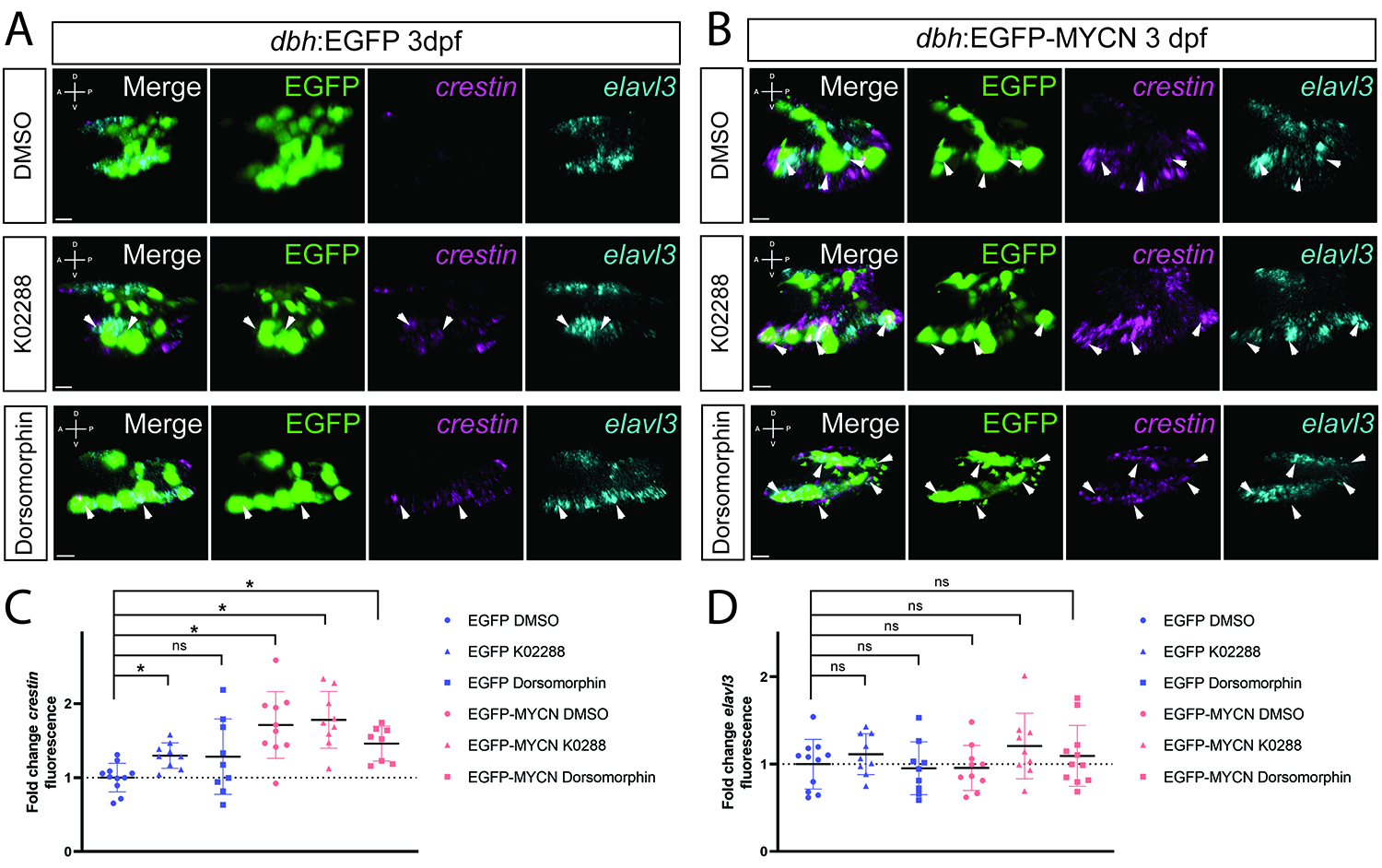

Supplement: S7 Fig — A,B) Representative images from developing SCG in dbh:EGFP (F) or dbh:EGFP-MYCN (G) larvae at 3 dpf after 24 h treatment with either DMSO (A,B, upper panels),K02288 (A,B, middle panels), or Dorsomorphin (A,B, lower panels). WICHCR performed using HCR probes against crestin, elavl3, and with antibody against EGFP. K02288 and Dorsomorphin treatments cause a discrete expansion of crestin expression in EGFP+ larvae. C,D) Mean crestin (C) and elavl3 (D) fluorescence intensity quantified in the SCG and normalized to dbh:EGFP average intensity at 3 dpf. For EGFP DMSO n = 11, EGFP K02288 n = 9, EGFP Dorsomorphin n = 9, EGFP-MYCN DMSO n = 10, EGFP-MYCN K02288 n = 9, EGFP-MYCN Dorsomorphin n = 8. F,G) Markers: EGFP (green), crestin (magenta), and elavl3 (cyan). Scale bar = 7 μm. For all graphs * P<0.05, n.s., non-significant (P>0.05). (TIF) [file pone.0310727.s007.tif]
